# DPY30 promotes colorectal carcinoma metastasis by upregulating ZEB1 transcriptional expression

**DOI:** 10.1186/s12935-023-03126-y

**Published:** 2023-12-19

**Authors:** Chun-Ying Luo, Wei-Chao Su, Hai-Feng Jiang, Ling-Tao Luo, Dong-Yan Shen, Guo-Qiang Su

**Affiliations:** 1 Medical College, Guangxi University, Nanning, 530004 Guangxi Province People’s Republic of China; 2 Fujian Psychiatric Center, Fujian Clinical Research Center for Mental Disorders, Xiamen Xianyue Hospital, Xianyue Hospital Affiliated With Xiamen Medical College, No. 55 Zhenhai Road, Xiamen, 361003 Fujian Province People’s Republic of China; 3 Department of Colorectal Tumor Surgery, School of Medicine, The First Affiliated Hospital of Xiamen University, Xiamen University, No. 55 Zhenhai Road, Xiamen, 361003 Fujian Province People’s Republic of China; 4 Xiamen Cell Therapy Research Center, School of Medicine, The First Affiliated Hospital of Xiamen University, Xiamen University, Xiamen, 361003 Fujian Province People’s Republic of China; 5 Department of Pathology, Affiliated Hospital of Youjiang Medical University for Nationalities, Baise, 533000 Guangxi Province People’s Republic of China

**Keywords:** DPY30, Colorectal carcinoma, EMT, Metastasis, H3K4me3, ZEB1

## Abstract

DPY30 belongs to the core subunit of components of the histone lysine methyltransferase complex, which is implicated in tumorigenesis, cell senescence, and other biological events. However, its contribution to colorectal carcinoma (CRC) progression and metastasis has yet to be elucidated. Therefore, this study aimed to investigate the biological function of DPY30 in CRC metastasis both in vitro and in vivo. Herein, our results revealed that DPY30 overexpression is significantly positively correlated with positive lymph nodes, epithelial-mesenchymal transition (EMT), and CRC metastasis. Moreover, DPY30 knockdown in HT29 and SW480 cells markedly decreased EMT progression, as well as the migratory and invasive abilities of CRC cells in vitro and lung tumor metastasis in vivo. Mechanistically, DPY30 increased histone H3K4me3 level and promoted EMT and CRC metastasis by upregulating the transcriptional expression of ZEB1. Taken together, our findings indicate that DPY30 may serve as a therapeutic target and prognostic marker for CRC.

**Graphical Abstract:**

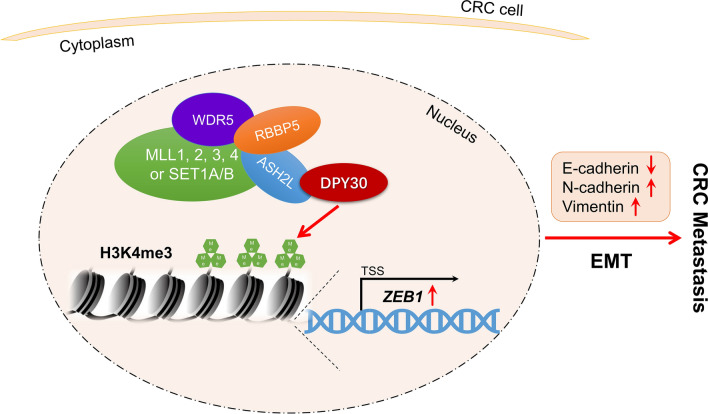

## Introduction

Colorectal carcinoma (CRC) is one of the most common gastrointestinal malignancies, currently ranks third in the incidence of malignant tumors worldwide, and is a leading cause of cancer-related mortality [1]. Its 5-year survival rate ranges between 10 and 90%, depending on the stage of the tumor [2]. Despite advances in CRC treatment strategies, including surgical and pharmacological interventions, improvements in survival outcomes remain limited [3, 4], particularly in patients with metastasis [5, 6]. Earlier studies have established a poor prognosis for patients with CRC metastases to lymph nodes or distant organs [7] Therefore, exploring the molecular mechanisms that drive metastasis holds considerable implications in promoting the development of novel diagnostic tumor markers and therapeutic strategies to enhance the prognosis of CRC patients.

Tumor metastasis is an intricate process that ultimately leads to cancer-related death. Meanwhile, an increasing number of studies have documented that epithelial-mesenchymal transformation (EMT) induction plays a key role in the transformation and development of cancer cells. The hallmarks of EMT encompass intercellular junctions, the absence of cell polarity, down-regulation of epithelial marker protein E-cadherin expression, which is essential for cell adhesion, and upregulation of the expression of interstitial marker proteins N-cadherin and vimentin, which are crucial the mesenchymal phenotype [8]. As is well documented, EMT is governed by transcription factors that can directly bind to the promoter of E-cadherin and inhibit its transcription, including Zinc finger protein family ZEB (ZEB1, ZEB2), Snail family (Snail1, Slug), and Twist family (Twist1, Twist 2) [9]. ZEB1 can directly inhibit the expression of E-cadherin, bind its zinc finger structure to the promoter e-box, and induce EMT [10]. Recent studies have revealed a link between EMT and chromatin configuration control caused by histone modifications [11, 12].

Tri-methylation of lysine 4 on histone H3 protein subunit (H3K4me3) is a major chromatin modification [13]. H3K4me3 exerts several biological effects and participates in processes such as cell differentiation [14] and tumorigenesis [15]. Of note, H3K4me3 modification promotes gene transcription by recruiting transcription factors and coactivators to the promoters and mediates H3K4me3 binding to the promoter region of genes [16]. H3K4me3 is catalyzed by SET/MLL COMPASS complex (SET/MLL COMplex of Proteins Associated with Set1), which consists of one of the six KMT2/MLL proteins (MLL1, 2, 3, 4, or SET1A/B). KMT2/MLL promotes mono-, di-, and tri-methylation of lysine 4 on histone H3 (H3K4me1, H3K4me2 and H3K4me3). The activity of the complex is largely contingent upon the binding of the catalytic subunit to the core subunit WRAD complex (including WDR5, RBBP5, ASH2L, and DPY30) [17].

DPY30 is a small protein (99 amino acids in humans) in the large SET1/MLL complex. While it does not directly bind to the catalytic subunit, it plays a decisive role in histone H3K4 methylation [18]. During embryonic stem cell development, components of the SET1/MLL complex participate in lysine methylation modification, gene transcription regulation [19], hematopoietic development [20], neural development [21], pancreas progenitor fate specification [22], and lifespan regulation [23]. DPY30 is critically implicated in cancers such as cholangiocarcinoma [24], MLL-rearranged leukemia [25], gastric cance r[26], and epithelial ovarian cancer [27]. Nevertheless, the expression level and clinical significance of DPY30 in CRC remain elusive. Moreover, the biological role of histone methylation regulated by DPY30 during EMT progression and metastasis remains underexplored. The difference is that the mechanism of DPY30 promoting cancer progression is different. Reports show that DPY30 can regulate cell proliferation and differentiation by affecting the expression of MYC oncoprotein and ID protein and the transcriptional activity of E2F. It has been found that the expression level of DPY30 can affect the EMT process in cervical squamous cell carcinoma [28]. Jeremy N. Rich reported that DPY30 regulated the expression of PDE4B in glioblastoma depending on H3K4me3, thus regulating angiogenesis and hypoxia pathways [29]. The effect of DPY30 on EMT of colorectal cancer cells to promote tumor metastasis has not been reported. The molecular mechanism that influences EMT by promoting ZEB1 by influencing histone methylation level in CRC has also not been reported.

Therefore, the current study aimed to investigate the biological function of DPY30 in CRC metastasis both in vitro and in vivo. Specifically, the correlations between DPY30 and the migratory and invasive abilities of CRC cells, as well as EMT and metastasis, were evaluated. Interestingly, DPY30 regulated EMT progress by targeting ZEB1 and affected the H3K4me3 histone. Our findings validated the vital role of DPY30 in CRC metastasis and suggest that it may serve as a potential diagnostic and therapeutic strategy for advanced CRC.

## Materials and methods

### Chemicals and antibodies

The following antibodies were used: DPY30 (Abacm, ab126352; ABclonal, A17796), GAPDH (CST, 5174), β-Actin, (CST, 4970), E-cadherin (CST, 3195), N-cadherin (CST, 13116), Vimentin (CST, 5741), Zeb1 (CST, 70512), Histone H3 (CST, 4499), H3K4me1 (CST, 5326), H3K4me2 (CST, 9725), and H3K4me3 (CST, 9751). Anti-mouse or rabbit secondary antibodies were sourced from Sigma. FBS, high glucose DMEM medium, and Trypsin (with EDTA) were purchased from Gibco (Thermo Fisher Scientific, USA). Penicillin–streptomycin was acquired from BasalMedia. Other reagents were all analytical grade.

### Stable cell lines and cell culture

The human CRC cell lines RKO, SW620, HCT116, SW1116, HT29, SW480, Caco2, and KM12C, as well as the normal colonic epithelial cell line NCM460, and 293 T cells were cultured in DMEM medium supplemented with 10% FBS, penicillin–streptomycin at 37 ℃ in a 5% CO_2_ cell incubator. The construction of DPY30 knockdown stable cell lines HT29 and SW480 cells followed the methodology outlined in previous research[24].

### Wound healing assay

A wound-healing assay was conducted to evaluate the migratory abilities of HT29 and SW480 cells. Cells were seeded in 6-well plates and allowed to attain a confluency of 70–90. Afterward, an artificial wound area was created using a 10 μL pipette tip. Then, the cells were washed with PBS and further incubated in a serum-free medium. Lastly, cell migration was visualized under a microscope (Leica DMi8, Germany), and images were captured at 0 h, 24 h, and 48 h.

### Cell invasion and migration assay

Cell motility was examined using 24-well plates with either uncoated inserts (8-μm pore, BD Biosciences) to evaluate migration or 20 μL Matrigel-coated inserts to evaluate invasion in vitro. Briefly, 1 × 10^5^ cells in 200 μL of FBS-free media were seeded in the upper chamber, while 700 μL of 20% FBS DMEM was placed in the lower chamber of the insert. After incubating for 24 and 48 h, respectively, the transwell was sequentially cleaned using a cotton swab, fixed with formaldehyde, stained with crystal violet, and photographed using an inverted phase contrast microscope (Leica DMi8, Germany).

### Patient and tissue specimens

Fifteen paired tissue samples, comprising CRC tumors, para-cancerous tissues, and distal healthy tissues, were randomly collected from CRC patients and used for western blot and immunohistochemistry analyses. They were all obtained through curative resection with informed consent from patients at the Department of Colorectal Tumor Surgery, Xiamen Cancer Hospital, First Affiliated Hospital of Xiamen University, Xiamen, China. This study was approved by the Ethics Committees of The First Affiliated Hospital of Xiamen University (Xiamen, China) (The IRB approval number: XMYY-2020KYSB040). All tissue samples were immediately collected and stored at −80 ℃ until further use. Besides, a tissue microarray (Shanghai Outdo Biotech Company (Cat No. HColA180Su19)) consisting of 94 CRC tissue sections was used for immunohistochemical analysis to determine the expression of DPY30 in patients with or without positive lymph nodes.

### Hematoxylin–eosin (H&E) staining

In short, the tissue samples were fixed with 10% paraformaldehyde overnight, subsequently dehydrated and embedded in paraffin by Automatic Benchtop Tissue Processor (Leica TP1020, USA), then sectioned at a thickness of 4 mm using a Manual Rotary Microtome (Leica RM2235, USA). After dewaxing and rehydration, the sections were stained with Mayer hematoxylin (5 min) and eosin (30 s) using a Hematoxylin–eosin Staining Kit (Baso, China).

### Immunohistochemical staining (IHC)

Tissues were fixed in 10% paraformaldehyde, followed by dehydration, paraffin embedding, and sectioning. After dewaxing using an environmentally friendly clearing agent, antigen retrieval was performed under high pressure in a pressure cooker by boiling the samples in citrate buffer. The sections were subsequently incubated in 3% H_2_O_2_ peroxidase blocking buffer for 10 min (Maxim Biotechnologies, China). Next, they were blocked with 10% donkey serum for 1 h. Tissue sections were incubated with the primary antibody overnight, followed by incubation with the secondary antibody. Finally, sections were visualized using a DAB Kit (Maxim Biotechnologies, China), followed by hematoxylin staining to locate the cells. IHC staining of protein in the tissue was scored according to the semi-quantitative immunoreactivity score [30].

### Western blotting assay

Briefly, cells or tissue samples were lysed with RIPA buffer (protease inhibitors and phosphatase inhibitors added). Protein concentration was determined using the BCA method (Thermo Fisher Scientific). Equal amounts of proteins were boiled, separated by 12% or 15% SDS-PAGE, and then electrotransferred to polyvinylidene difluoride membranes (Roche) using the eBlot^™^ L1 wet protein transfer system (GenScript). After blocking with 5% milk, the membranes were incubated with primary antibodies overnight and subsequently incubated with secondary antibodies. An ultra-sensitive Enhanced Chemiluminescence Substrate Kit (Biothrive) was used to detect the immunoreactive substrates.

### RT-qPCR assay

The cells or tissue samples were collected, and total RNA was extracted using the RNAsimple Total RNA Kit (related all obtained from Tiangen Biotech). Then, RNA was reverse transcribed to cDNA using a FastQuant RT Kit (with gDNase). qPCR was prepared using the SuperReal PreMix Plus kit with specific primers (Table. 1) and performed on an ABI 7500 real-time PCR machine. The obtained cycle threshold number (Ct value) was normalized utilizing the 2^−ΔΔCT^ method. All Ct values were normalized using the Ct value of β-actin as the reference gene.

**Table 1:** Primers used for gene expression detection

Gene	Primer forward (5ʹ–3ʹ)	Primer reverse (5ʹ–3ʹ)
RT-qPCR primers		
DPY30	AACGCAGGTTGCAGAAAATCCT	TCTGATCCAGGTAGGCACGAG
E-Cadherin	TGAAGGTGACAGAGCCTCTGGAT	TGGGTGAATTCGGGCTTGTT
N-Cadherin	AGATAGCCCGGTTTCATTTGAG	ATGTTGGGTGAAGGGGTGCT
Vimentin	AATGGCTCGTCACCTTCGTG	CAGATTAGTTTCCCTCAGGTTCAG
Fibronectin	CACTTCAGTGGGAGACCTCGAG	GGTCCCTCGGAACATCAGAAAC
Snail1	GCTACTGCTGCGCGAATCG	GTAGGGCTGCTGGAAGGTAAACT
Slug	TTCGGACCCACACATTACCTTG	ACCTGTCTGCAAATGCTCTGTTG
Twist1	CGGCTCAGCTACGCCTTCT	CAATGACATCTAGGTCTCCGGC
Twist2	CATGTCCGCCTCCCACTAGC	ATGTGCTCACTCCCGCCAAC
Zeb1	AGGCTATAAACGCTTTACCTCTCTG	TTACGATTACACCCAGACTGCGT
β-actin	CATGTACGTTGCTATCCAGGC	CTCCTTAATGTCACGCACGAT
ChIP-qPCR primers		
#1 Zeb1	AAATTCAGCAGTGCCCACG	TTACGACACTCCCGGCTTTAC
#2 Zeb1	GGCAAAGTGGAGTGGGAAAG	AGACATAACGGTTCAGGGAGA
#3 Zeb1	TCTTACCTGGTCTCTCTCCGC	AGAAAAGTGGCCAGTGCGT

### Chromatin immunoprecipitation assay (ChIP)-qPCR

ChIP assay was performed using a SimpleChIP^®^ Plus Sonication Chromatin IP Kit (#56383, CST) following the manufacturer's instructions. HT29 and SW480 cells were cross-linked in 1% formaldehyde and lysed. Chromatin fragments were acquired via sonication (200 ~ 1000 bp). ChIPs were incubated with anti-H3K4me3 antibody or anti-rabbit IgG overnight. The complexes were precipitated using Protein G Magnetic Beads for 2 h. Next, the protein-DNA cross-links were reversed, and DNA was purified. DNA sequences for ZEB1 were analyzed using qPCR using an ABI-7500 System (Applied Biosystems) and SuperReal PreMix Plus (Tiangen Biotech). IP efficiency was manually presented as the percent input method, calculated using the following equation: Percent Input** = **2% × 2^(C[T] 2% Input Sample − C[T] IP Sample)^.

### Animal models of tumor metastasis and MicroPET/CT imaging

Nude mice were obtained from the Xiamen University Laboratory Animal Center. The animal experiments were approved by the Animal Care and Use Committee of Xiamen University and undertaken according to the institution's guidelines. Regarding tumor metastasis assays, the cells were resuspended in PBS, and mice were injected slowly via the tail vein with 1 × 10^7^ DPY30-knockdown or control cells, which included HT29 and SW480 cells (n = 7 per group). Nude mice were monitored using PET/CT scanning in the 8th week. After 10 weeks, the mice were euthanized, and lung tissues were dissected and collected. The number of metastatic nodules in the lung parenchyma was counted. Lastly, lung sections were fixed and stained with H&E staining.

A MicroPET/CT (Siemens Inveon, USA) was used for PET/CT scanning and image analysis of pulmonary tumor metastasis. Each nude mouse was anesthetized with isoflurane and then injected with 200 μCi of 18^F^-FDG through the tail vein. Three-dimensional ordered-subset expectation maximization (3D-OSEM)/maximum algorithm was employed for image reconstruction to generate transverse and coronal sections of CT images and PET-CT overlapped images.

### Statistical analysis

Statistical analyses were conducted using GraphPad Prism version 8.0.1. The data were presented as the mean ± SEM. The two-tailed Student's *t*-test was used to compare differences between the two groups. Comparisons among groups were performed using analysis of variance (either one-way or two-way ANOVA as appropriate). *P < 0.05, **P < 0.01, and ***P < 0.001 were considered statistically significant.

## Results

### DPY30 overexpression in CRC samples was correlated with EMT and tumor metastasis

**Fig. 1:**
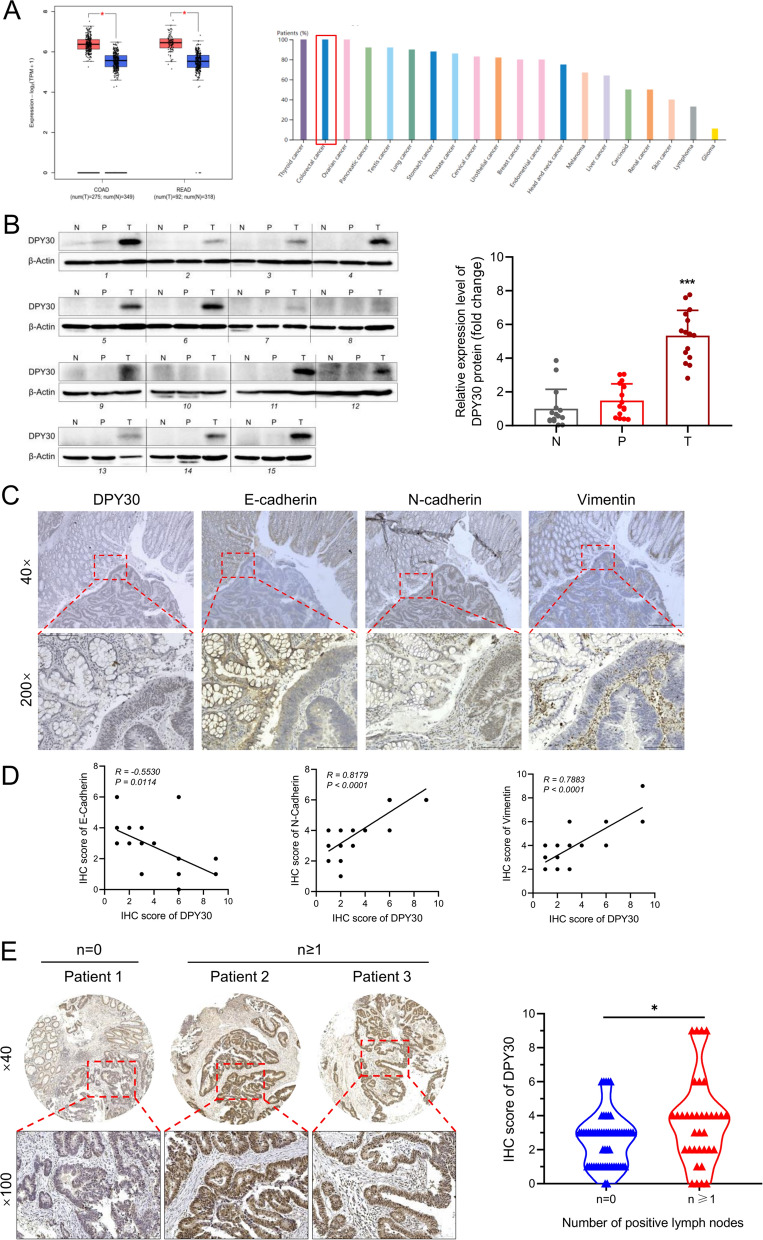
Overexpression of DPY30 is correlated with EMT and tumor metastasis in human colorectal carcinoma. **A** Analysis of DPY30 expression profile across tumor samples and paired healthy tissues using the GEPIA online database, and the tissue-wise expression of DPY30 in different cancer types using The Human Protein Atlas website. **B** DPY30 protein expression in the paired tissue samples was detected by western blot (N: distal healthy tissues. P: para-cancerous tissues. T: CRC tumor tissues). Comparison of the relative protein expression levels of DPY30, compared with N and P (n = 15). Data are expressed as mean ± SEM. ***P < 0.001. **C** Expression levels of DPY30, E-Cadherin, N-Cadherin, and Vimentin in tumor in situ and para-tissue by immunohistochemical staining analysis. **D** Correlation scatter plot of the IHC score of DPY30 and the EMT markers E-Cadherin, N-Cadherin, and Vimentin, respectively; (n = 15). **E** Representative immunostaining images of DPY30 in a CRC tissue microarray from patients with or without positive lymph nodes (Number of patients = 94), along with attached scores displaying the correlation of DPY30 immunostaining intensity with the number of positive lymph nodes. Data are presented as mean ± SEM. *P < 0.05

To investigate the role of DPY30 in human colorectal carcinoma (CRC), the expression levels of DPY30 in CRC tissues, para-cancerous tissues, and paired healthy tissues were determined. Online dataset analysis of the GEPIA database (http://gepia.cancer-pku.cn/index.html) indicated that the expression level of DPY30 was higher in colon adenocarcinoma (COAD) and rectal adenocarcinoma (READ) than in healthy tissues (*P* < 0.05) (Fig. 1A, left). Additionally, the tissue-wise expression of DPY30 in different cancer types using The Human Protein Atlas website revealed that DPY30 was the second most highly expressed gene in CRC compared with other cancers. (www.proteinatlas.org) (Fig. 1A, right). Meanwhile, the DPY30 expression level in CRC tissues (T) was significantly higher compared to that in para-cancerous tissues (P) and paired healthy tissues (N) (n = 15; *P* < 0.001) (Fig. 1B). In our previous studies [31]. elevated DPY30 protein levels positively correlated with clinicopathological characteristics, such as pathological grading, tumor size, TNM stage in CRC patients through tissue microarray. On this basis, the correlation between the expression of DPY30 and EMT was further assessed in a sample of 15 CRC patients with positive lymph nodes. As illustrated in Fig. 1C, the expression of DPY30, N-Cadherin, and Vimentin were upregulated in the CRC tumor region (bottom right), but the expression level of E-Cadherin in the CRC tumor region (bottom right) was lower than that in para-tissues (upper left). Interestingly, analysis of the correlation scatter plot of the IHC score exposed that DPY30 was negatively correlated with E-Cadherin (*R* = −0.5530, *P* = 0.0114) and positively correlated with N-Cadherin (*R* = 0.8179, *P* < 0.001) and Vimentin (*R* = 0.7883, *P* < 0.001) (Fig. 1D). Furthermore, the expression level of DPY30 in CRC patients with positive lymph nodes (number, n ≥ 1) was significantly higher than in patients without positive lymph nodes (n = 0) (*P* < 0.05) (Fig. 1E). These data collectively demonstrated that the upregulation of DPY30 expression might be correlated with EMT and CRC metastasis.

### DPY30 knockdown suppressed EMT progression in CRC cells

**Fig. 2:**
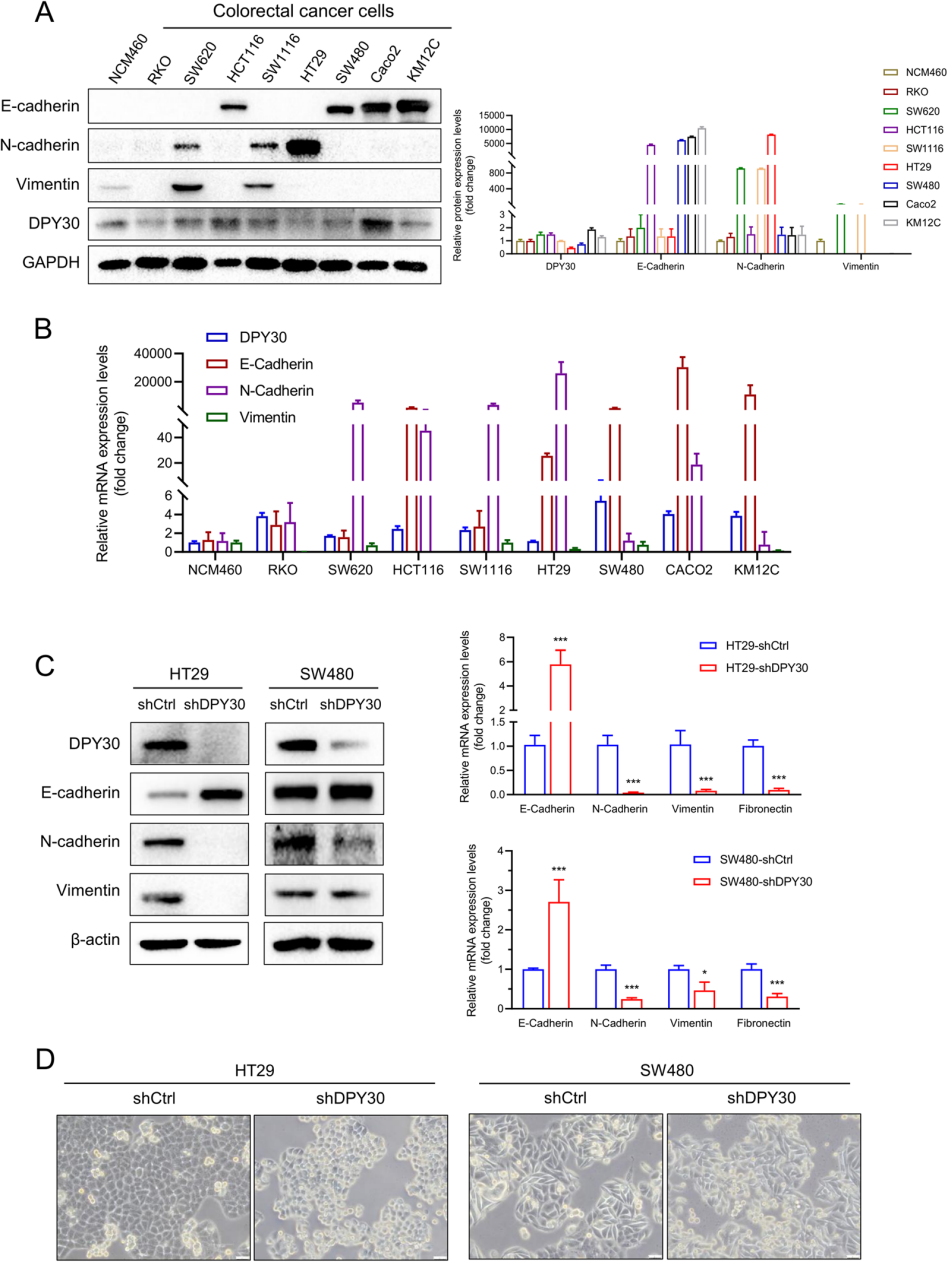
Knockdown of DPY30 inhibits EMT in CRC cells. **A** Western blotting analysis of DPY30 and EMT markers in CRC cells RKO, SW620, HCT116, SW1116, HT29, SW480, Caco2, KM12C, and the normal colonic epithelial cell line NCM460 (left). Densitometry quantifications were performed using Image Lab software (right). **B** Quantitative real-time PCR analysis of the mRNA levels of DPY30 and EMT in CRC cells. **C** Western blotting (left) and qPCR (right) analysis of DPY30 and EMT markers in DPY30-knockdown CRC cell lines. Data are expressed as mean ± SEM of three independent experiments. *P < 0.05, ***P < 0.001. **D** Representative phase-contrast images of HT29 and SW480 cells showing morphological alterations following DPY30 knockdown

Based on the correlation between DPY30 expression and its correlation with CRC metastasis, further experiments were carried out at the cellular level. Western blotting and qPCR results validated that the expression level of DPY30 was higher in RKO, SW620, HCT116, SW1116, HT29, SW480, Caco2, and KM12C cells (Fig. 2A, B). Furthermore, the expression of the epithelial gene E-cadherin was inversely correlated with DPY30 levels, especially in HT29 cells, while the expression of the mesenchymal gene N-cadherin was positively correlated with DPY30 in HT29 cells, signaling that HT29 cells may manifest a more mesenchymal phenotype compared to other cells. Based on the comprehensive consideration through two dimensions results: Western Blot and qPCR. Moreover, many studies have used two colorectal cancer cell lines, HT29 and SW480, as experimental research objects to study the role and mechanism of regulating EMT in CRC [32–37]. It indicates that these two cell lines are suitable and have important roles and research significance for CRC EMT and metastasis. Therefore, we finally selected these two cell lines to further explore the mechanism of DPY30 on EMT. Therefore, retroviruses carrying short hairpin RNA (shRNA) targeting DPY30 were introduced into HT29 and SW480 cells to generate HT29-shDPY30 and SW480-shDPY30 stable cell lines (Fig. 2C). Notably, HT29-shDPY30 and SW480-shDPY30 cells exhibited a fibroblastic morphology (Fig. 2D). This observation further corroborated that DPY30 knockdown increased the expression level of the epithelial marker E-cadherin and decreased that of mesenchymal markers (N-cadherin, vimentin, and fibronectin) (Fig. 2C). These findings conjointly suggested that DPY30 facilitates the transition between epithelial to mesenchymal phenotypes and plays an instrumental role in regulating the EMT plasticity of CRC cells.

### DPY30 knockdown decreased the migratory and invasive capacities of CRC cells in vitro

**Fig. 3:**
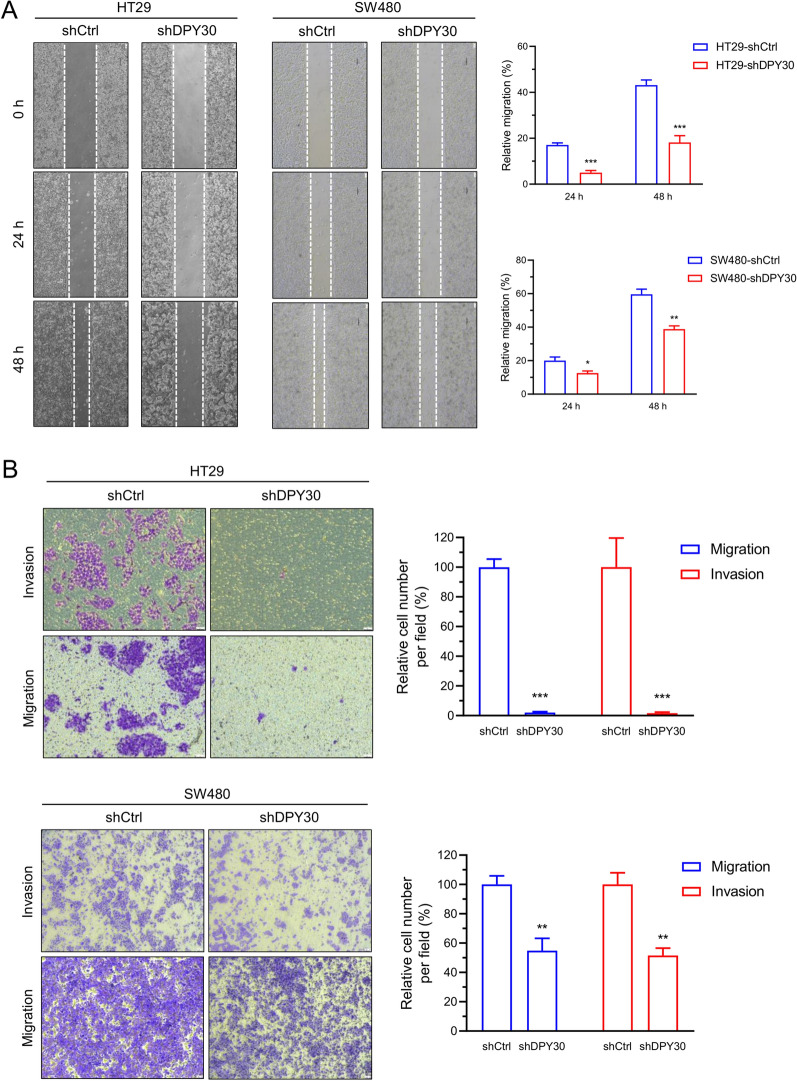
Knockdown of DPY30 decreases the migratory and invasive capacities of CRC cells in vitro. HT29-shDPY30 and SW480-shDPY30 cells or their control cells were subjected to wound healing assay (**A**), Transwell migration (**B**, top), and Matrigel invasion assays (B, bottom) for 24 h and 48 h, respectively. **A** Effects of DPY30 knockdown on the migratory capabilities of CRC cells. The uncovered areas in the wound healing assays were quantified as a percentage of the original wound area. Statistical analysis of A, right. **B** Effects of DPY30 knockdown on the migratory and invasive abilities of CRC cells. Quantification of migrated cells through the membrane and invaded cells through Matrigel are presented as proportions relative to their controls. Statistical analysis of B, right. Data are presented as mean ± SEM of three independent experiments. *P < 0.05, **P < 0.01, ***P < 0.001

Cancer metastasis is frequently the leading cause of anti-tumorigenic treatment failure, which is typically accompanied by EMT. DPY30 has been postulated to affect the migratory and invasive capabilities of CRC cells. As displayed in Fig. 3, transwell migration and matrigel invasion assays determined that the migratory and invasive abilities of HT29-shDPY30 (*P* < 0.001) and SW480-shDPY30 (*P* < 0.01) were decreased compared with HT29-shCtrl and SW480-shCtrl, respectively. (Fig. 3B). Moreover, DPY30 knockdown cells had a significant faster closure of the wound area compared with control CRC cells at 48 h (HT29, *P* < 0.001; SW480, *P* < 0.01). (Fig. 3A). The aforementioned results indicated that DPY30 is significantly correlated with the migratory and invasive abilities of CRC cells and may promote cell migration and invasion by promoting EMT changes in CRC cells.

### DPY30 regulated ZEB1 by attenuating H3K4me3 binding to the promoter region of ZEB1

**Fig. 4:**
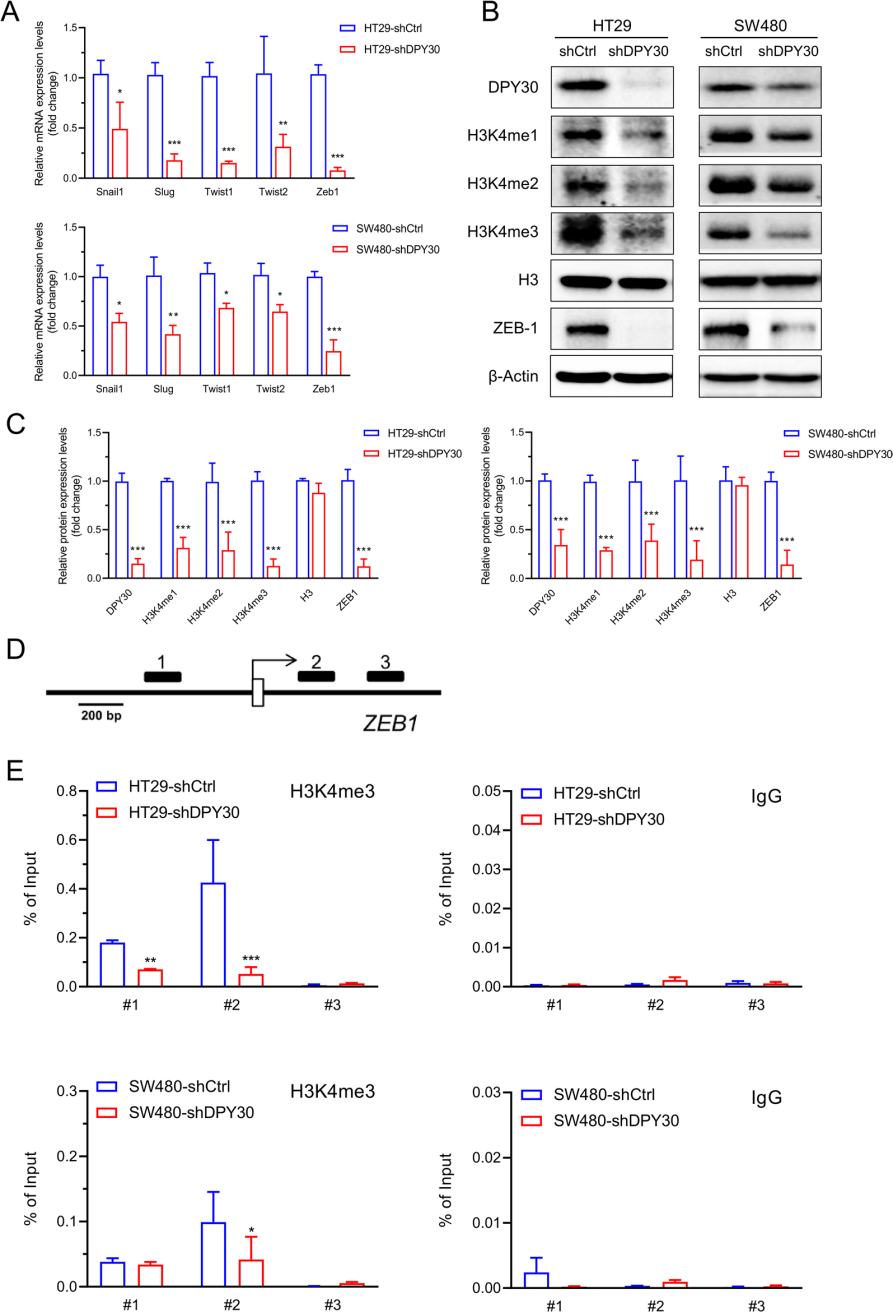
DPY30 promotes ZEB1 transcriptional expression through H3K4 methylation and regulates H3K4me3 binding to the promoter region of ZEB1 in CRC cells. **A** mRNA levels of EMT-related regulatory factors Snail1, Slug, Twist1, Twist2 and Zeb1 in CRC cells with DPY30 knockdown as measured by RT-qPCR. **B** The expression level of ZEB1 and H3 lysine methylation levels were assessed in CRC cells following DPY30 knockdown by Western blotting using whole-cell lysate; total H3 and β-actin were used as loading controls. **C** The intensity quantified statistical analysis of WB results. **D** Schematic presentation of the three regions relative to the ZEB1 transcriptional start site used as primers to test H3K4me3 occupancy. **E** ChIP-qPCR was performed to assess H3K4me3 occupancy at the ZEB1 transcriptional start site in HT29-shDPY30, SW480-shDPY30, and their control cells (**E**, left). IgG was used as a negative control (**E**, right). "Percentage of input" represents the ratio of DNA fragments of each promoter region bound by H3K4me3 to the total amount of input DNA fragments without H3K4me3 antibody pull-down. Data are expressed as mean ± SEM of three independent experiments. *P < 0.05, **P < 0.01, ***P < 0.001

According to the above-mentioned results at the clinical and cellular levels, the mechanism by which DPY30 promotes EMT was further evaluated. As depicted in Fig. 4, RT-qPCR results showed that the expression levels of EMT-related transcription factors Snail1, Slug, Twist1, Twist2, and Zeb1 were significantly down-regulated in DPY30 knockdown cells (Fig. 4A). Furthermore, western blot analysis revealed a significant decrease in the protein expression level of ZEB1 protein in DPY30 knockdown cells (Fig. 4B). In addition, the modulated histone modification pattern was determined. Noteworthily, the expression of H3K4me1, H3K4me2, and H3K4me3 were also significantly decreased (Fig. 4B, C). Next, the correlation between DPY30 expression and H3K4me3 modification at the ZEB1 promoter region was assessed. Three pairs of primers at the promoter region of the ZEB1 gene were designed and synthesized (Fig. 4D). ChIP-qPCR determined decreased occupancy of ZEB1 gene promoter regions by H3K4me3 in shDPY30 cells (Fig. 4E). Besides, DPY30 knockdown attenuated H3K4me3 levels in region −1413 to −1294 bp (#1, *P* < 0.01) and + 188 to + 294 bp (#2,* P* < 0.001) of the ZEB1 gene promoter in HT29-shDPY30 cells, as well as in region + 188 to + 294 bp (#2) in SW480-shDPY30 cells (*P* < 0.05). Overall, these results indicated that DPY30 knockdown induces transcriptional inactivation of ZEB1 by regulating H3K4me3 and decreasing H3K4me3 levels at the ZEB1 gene promoter region.

### Downregulation of DPY30 suppressed CRC cell metastasis in vivo

**Fig. 5:**
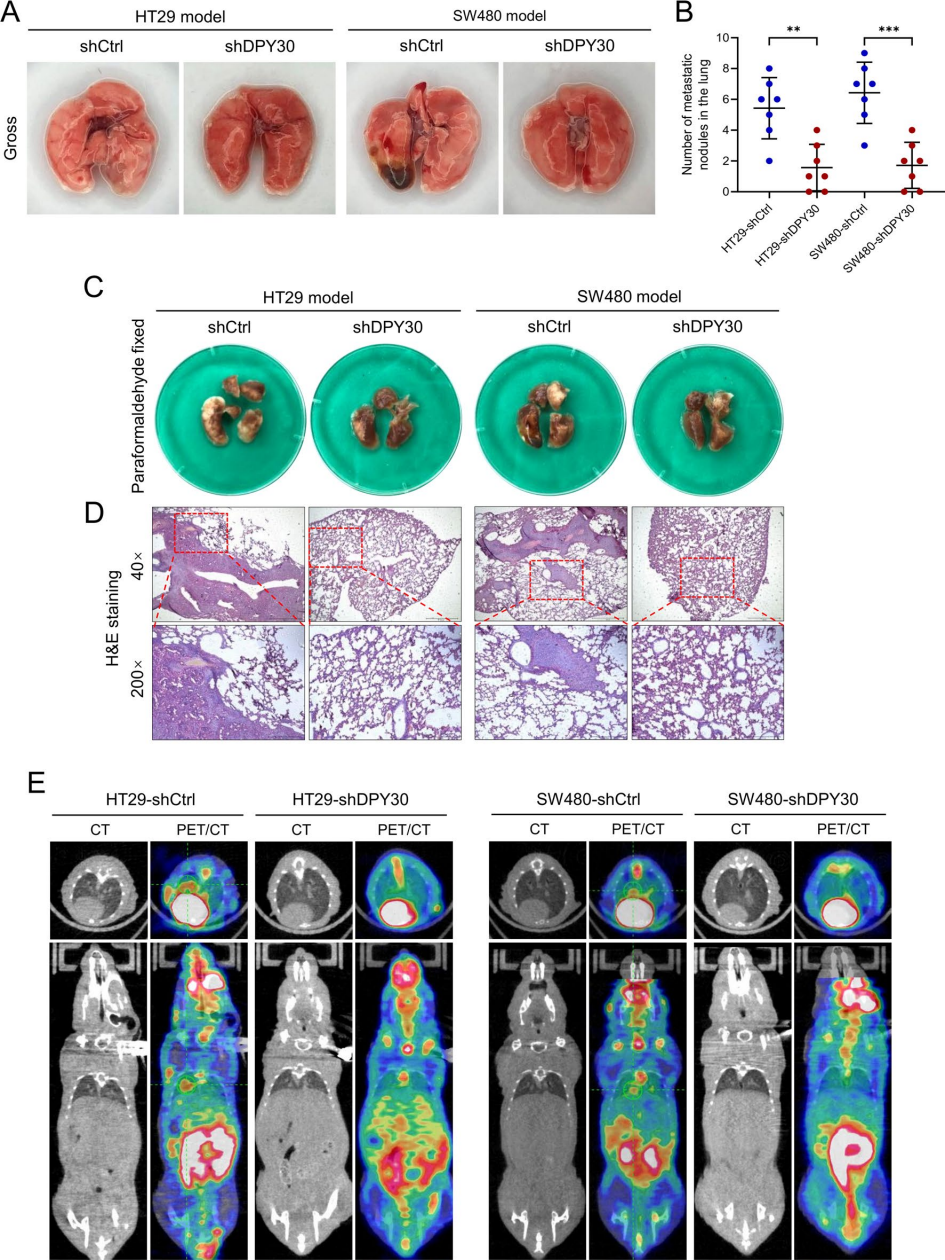
Downregulation of DPY30 suppresses CRC tumor metastasis in vivo*.* **A** Representative gross images of lung metastatic nodules in nude mice that received intravenously tail vein injection of HT29 and SW480 cells with stable DPY30 knockdown (HT29-shDPY30 and SW480-shDPY30) or control vector at 10 weeks. **B** Number of metastatic tumor nodules in the lung of mice. Data are presented as mean ± SD; n = 7 for each group. **P < 0.01, ***P < 0.001. **C** Representative images of lung tissues from mice after fixing with 10% paraformaldehyde. **D** Representative images of hematoxylin and eosin-stained histological sections of lungs from nude mice with/without metastasis illustrating local invasiveness. (Left, HT29 model; right, SW480 model). **E** Representative images of transverse (upper) and coronal (bottom) sections of CT images and PET-CT images of nude mice compared with corresponding controls

To confirm our in vitro observations and investigate the function of DPY30 in migration, invasion, and metastasis in vivo, animal models of tumor metastasis were established by injecting HT29-shDPY30 or SW480-shDPY30 cells and their corresponding control cells into nude mouse tail veins. Afterward, small-animal MicroPET/CT scanning was performed to evaluate the pulmonary colonization of these cells. As anticipated, PET-CT images displayed spherical metastatic nodules in the lung parenchyma in the shDPY30 group (Fig. 5E). Moreover, mice injected with HT29 and SW480 cells carrying shDPY30 had a significantly lower number of lung metastatic nodules (HT29 group, P < 0.01; SW480 group, P < 0.001) (Fig. 5A, B), implying that silencing DPY30 in HT29 and SW480 cells inhibited metastatic behavior both in terms of the number of distant pulmonary metastatic nodules and the severity of metastatic pulmonary tumors (Fig. 5C, D). Therefore, the in vivo results showcased the critical role of DPY30 in CRC metastasis.

## Discussion

CRC is a global health concern owing to its mortality and morbidity rates among the general population. EMT is implicated in the development and metastasis of several tumors, including CRC [38]. Our results suggest that DPY30 plays a vital role in promoting EMT progression in CRC cells and in facilitating tumor metastasis. It also regulates CRC metastasis through regulating histone modification H3K4me3 methylation and ZEB1 transcriptional expression.

DPY30 is a relatively understudied member of the SET1/MLL histone methyltransferase complex. Histone methyltransferase is involved in histone modification and plays an essential role in tumor progression. Members of the SET1/MLL protein family, including core subunits and catalytic subunits, act as transcriptional regulators and contribute to disease onset [39]. The mechanism of action of the SET1/MLL family in tumors is intricate and poorly understood. Members of the Set1 complex are commonly referred to as "WRAD", which stands for WDR5, RBBP5, ASH2L, and DPY30, and these core subunits are required for histone methylation activity [40, 41]. For instance, WDR5 is abundantly expressed in prostate cancer and conduces to androgen-induced tumor cell proliferation [42]. ASH2L can interact with the MYC oncoprotein to induce the transformation of MYC/HaRAS-dependent fibroblasts in rat embryos [43]. Meanwhile, ASH2L overexpression can also significantly contribute to tumor proliferation [44]. Based on the prior studies on the functions of the same type of core subunits, we hypothesize that DPY30 plays a similar carcinogenic role in CRC. Further investigations are warranted to explore the interactions between DPY30 and other core subunits in order to elucidate their role in promoting cancer metastasis.

DPY30 has been reported to promote cell proliferation and regulate cell cycle [20, 25, 26]. A previous study pointed out that DPY30 knockdown in SKOV3 cells up-regulated the expression of E-cadherin and down-regulated that of vimentin, N-cadherin, and Snail [27], demonstrating the potential tumorigenic and metastatic effects of DPY30. However, the correlation of DPY30 with tumor metastasis and the mechanism by which it triggers metastasis remain to be elucidated. This study provides compelling evidence that DPY30 is significantly overexpressed in colorectal cancer tissues, with higher expression levels observed in cases with lymph node metastasis compared with those without lymph node metastasis. To the best of our knowledge, this is the first study to confirm that DPY30 is associated with EMT and induces metastasis via clinical, cellular, and animal metastasis models rather than solely focusing on the mechanism by which DPY30 promotes the proliferation and growth of CRC cells. Importantly, alterations in ZEB1 transcriptional expression levels were found to be involved in the regulation of histone methylation. Notwithstanding, the luciferase reporter gene assay is necessary to identify the interactions between DPY30, ZEB1, and specific promoter sites in CRC cells. Overall, the present study highlighted the potential application of DPY30 as a therapeutic target for CRC.

Furthermore, DPY30 can interact with ASH2L and affect the conformation of DPY30, but its interaction with the other three subunits has not been reported. And the mechanism of DPY30 promoting CRC metastasis through its interaction with ASH2L has not been reported, which needs further study. It has been reported that the lipolysis factor ABHD5 can interact with DPY30 in the cytoplasm, thereby inhibiting the nuclear translocation of DPY30 and reducing the activity of SET1A and the dryness of CRC cells [45]. As for whether there any potential interactions between DPY30 and other core subunits of the SET1/MLL complex that contribute to its effects on CRC metastasis, we think it worth further study. We mainly focus on the mechanism by which DPY30 affects histone H3K4me3 levels and ZEB1 transcriptional expression in CRC, thereby promoting EMT and CRC metastasis.

EMT is responsible for cell reprogramming that enables tumor cells to acquire certain new abilities, which in turn reshape the relationship between cells and the tumor microenvironment [46]. Herein, the expression level of DPY30 was significantly correlated with the expression level of EMT markers, both in clinical tissue IHC detection results and in the phenotypic changes in CRC cells following DPY30 knockdown. This finding insinuates that DPY30 participates in EMT. Meanwhile, up-regulation of ZEB1 is closely related to the subsequent metastasis of EMT in CRC. At the same time, Western blot unveiled that DPY30 knockdown significantly down-regulated the expression of EMT-related transcription factors, especially ZEB1 and H3K4me3. In addition, the mRNA expression level of ZEB1 was significantly altered, as presented in Fig. 4 (*P* < 0.001). We speculate that the effect of DPY30 in CRC may also be associated with ZEB1. The results of the ChIP assay support the hypothesis that DPY30 is involved in CRC metastasis by promoting ZEB1 transcriptional expression through regulating histone H3K4 methylation. Regarding the overexpression of DPY30 in many cancers, the reports related to the change of H3K4 methylation mainly involve the role of DPY30 in promoting the proliferation of tumor cells, and few studies have been conducted on the role of DPY30 in promoting EMT, and the mechanism of promoting EMT is lacking. For example, the previous study about DPY30 regulating cervical squamous cell carcinoma by mediating EMT [28], and DPY30 is required for the enhanced proliferation, motility and EMT of epithelial ovarian cancer cells [27]. In this study, the functional mechanism of DPY30 is different from that of reported studies. It has not been reported that DPY30 affects the CRC EMT and tumor metastasis. The innovation of this study is the molecular mechanism by which DPY30 in CRC promotes ZEB1 by influencing the level of histone methylation and thus affects EMT.

## Conclusions

**Fig. 6:**
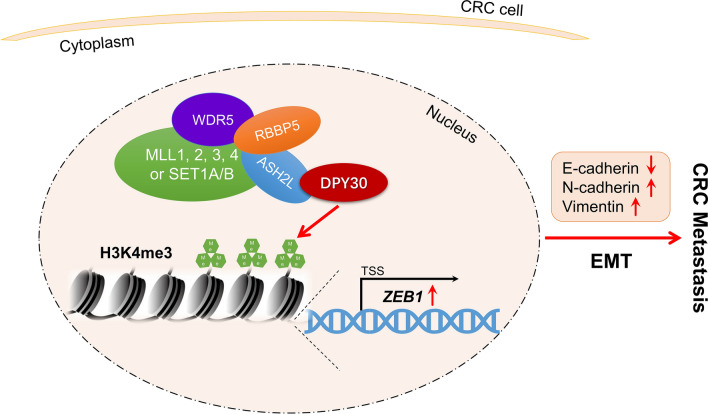
Schematic model illustrating the role of DPY30 in the regulation of EMT progression and CRC metastasis. DPY30 may upregulate histone H3K4me3 modification on the ZEB1 promoter and eventually boost the migratory and invasive abilities of CRC cells and facilitate EMT progression, leading to CRC metastasis

Taken together, the current study demonstrated the important role of DPY30 in facilitating EMT and CRC metastasis (Fig. 6). DPY30 enhanced the migratory and invasive abilities of CRC cells and EMT progression, leading to CRC metastasis by upregulating ZEB1 expression through histone H3K4me3 modification. Thus, DPY30 may represent a therapeutic target and prognostic marker for CRC.

## Data Availability

All data generated or analyzed during this study are presented in this published article. The data that support the findings are available from the corresponding author upon reasonable request.

## Abbreviations

CRC: Colorectal carcinoma
EMT: Epithelial-mesenchymal transition
COMPASS: SET/MLL complex of proteins associated with set1
ZEB1: Zinc Finger E-Box Binding Homeobox 1
H3K4me1: Mono-Methylation-Histone H3 (Lys4)
H3K4me2: Di-Methylation-Histone H3 (Lys4)
H3K4me3: Tri-Methylation of lysine 4 on histone H3 protein subunit
RT-qPCR: Quantitative Real-time Polymerase Chain Reaction
ChIP: Chromatin immunoprecipitation
